# Milk-Derived Extracellular Vesicles Protect Bovine Oviduct Epithelial Cells from Oxidative Stress

**DOI:** 10.3390/cells15010018

**Published:** 2025-12-22

**Authors:** Seyed Omid Reza Mousavi, Qurat Ul Ain Reshi, Kasun Godakumara, Subhashini Muhandiram, Getnet Midekessa, Aneta Andronowska, Sergei Kopanchuk, Darja Lavogina, Ago Rinken, Suranga Kodithuwakku, Alireza Fazeli

**Affiliations:** 1 Institute of Veterinary Medicine and Animal Sciences, Estonian University of Life Sciences, 51006 Tartu, Estonia; 2 Department of Pathophysiology, Institute of Biomedicine and Translational Medicine, Faculty of Medicine, University of Tartu, 14B Ravila, 50411 Tartu, Estonia; 3 Institute of Animal Reproduction and Food Research, Polish Academy of Sciences, 10-748 Olsztyn, Poland; 4 Institute of Chemistry, University of Tartu, 50411 Tartu, Estonia; 5 Department of Animal Science, Faculty of Agriculture, University of Peradeniya, Peradeniya 20400, Sri Lanka; 6 Division of Clinical Medicine, School of Medicine & Population Health, University of Sheffield, Sheffield S10 2SF, UK

**Keywords:** extracellular vesicles, oxidative stress, epithelial cell, miRNA

## Abstract

**Highlights:**

**What are the main findings?**

- Milk extracellular vesicles (mEVs) attenuate CoCl_2_-induced cytotoxicity and promote cell migration in bovine oviduct epithelial cells (BOECs).
- Analysis of mEV miRNA and protein cargo revealed biological pathways that facilitate cellular recovery from oxidative stress (OS).

**What are the implications of the main findings?**

- The protective effects of mEVs against CoCl_2_-induced stress in BOECs are likely mediated by their miRNA and protein cargo, providing a foundation for future mechanistic studies.
- mEVs may hold potential as a therapeutic approach for alleviating oviduct-associated OS.

**Abstract:**

Extracellular vesicles (EVs) are promising therapeutic agents due to their role in intercellular communication. This study examined the protective effects of milk-derived EVs (mEVs) on bovine oviductal epithelial cells (BOECs) under cobalt chloride (CoCl_2_)-induced oxidative stress (OS), comparing EVs stored at −80 °C or lyophilized. mEVs and algae-derived EVs (aEVs; negative control) were isolated via tangential flow filtration and applied at 10^7^, 10^9^, and 10^11^ particles/mL in three treatment strategies: pre-treatment, co-incubation, and post-treatment. mEVs specifically enhanced cell viability in all protocols except for post-treatment, where only 10^7^ particles/mL was effective; meanwhile, storage method did not affect EV activity. Enzyme digestion suggested that internal EV cargos are potentially the dominant contributors to the protective response compared to surface-associated molecules. mEVs reduced the expression of the OS markers *DDIT4* and *HIF1A* while promoting cell migration more effectively than aEVs. Pathway enrichment analysis of previously reported mEV miRNAs indicated regulation of cytokine production and glucocorticoid responses, potentially contributing to OS defense. mEV protein cargo analysis showed pathways primarily linked to peptidase and vesicle-related functions, suggesting that protein cargo may also contribute to the observed protective effects. Overall, mEVs protect BOECs against CoCl_2_-induced OS and maintain bioactivity after lyophilization.

## 1. Introduction

The oviduct is a crucial reproductive organ that links the ovary to the uterus and serves as the key site for transportation and the final maturation of gametes [1], fertilization, and early embryonic development in humans and other mammals [2,3,4]. The proper execution of these reproductive functions strongly depends on the oviductal microenvironment, where maintaining a delicate balance between reactive oxygen species (ROS) and antioxidants is crucial for sustaining optimal conditions [5,6]. ROS in the oviduct can be generated from external environmental factors as well as from internal cellular processes within spermatozoa, oocytes, and embryos as byproducts of cellular energy metabolism [1,7].

At physiological levels, ROS play a beneficial role in supporting fertilization and early embryogenesis; however, excessive ROS production leads to oxidative stress (OS), which is cytotoxic [8] and has been associated with various reproductive disorders [9]. For example, hydrogen peroxide, a well-known oxidizing agent, has been shown to impair bovine sperm motility in vitro [10], while elevated ROS levels by lipid peroxidation reduce sperm penetration into oocytes and disrupt sperm-egg fusion in humans [11]. Oviductal cells are particularly enriched with antioxidants [12], providing a potential defense mechanism against OS. Previously, diverse enzymatic antioxidants present in the cow oviduct have been reported [13]. Additionally, it has been shown that membrane proteins from human oviductal OE-E6/E7 cells protect spermatozoa against OS by reducing lipid peroxidation and ROS levels [14].

Alternatively, exogenous antioxidant supplementation has been explored as an interventional approach to mitigate OS. For instance, improved cell viability and lower levels of lipid peroxidation have been reported in stressed bovine oviduct epithelial cells (BOECs) treated with antioxidant astaxanthin in vitro [15]. Moreover, supplementing the diet of cattle under heat stress conditions with antioxidants can reduce OS, which could indirectly benefit the reproductive system [16].

Recent research has highlighted the therapeutic potential of extracellular vesicles (EVs) in mitigating OS, particularly those derived from plants and certain animal cells, due to their intrinsic antioxidant properties [17]. Milk-derived EVs (mEVs), in particular, possess notable regenerative abilities owing to their rich composition of growth factors, antioxidants, and immunomodulatory substances [18,19]. While research on the therapeutic applications of mEVs is advancing rapidly, their potential to alleviate OS-induced oviductal dysfunction remains largely unexplored.

Therefore, the current study aimed to explore the protective effects of mEVs on BOECs under CoCl_2_-induced OS. CoCl_2_ is a chemical inducer of ROS that drastically reduces cell viability by damaging DNA, proteins, and lipids, ultimately causing apoptosis [20]. Therefore, we evaluated the ability of mEVs to restore the viability of BOECs under OS. Algae-derived EVs (aEVs) were used as negative controls to ensure that the observed functional effects were specific to mEVs rather than a general feature of EVs. Both frozen and lyophilized EVs were used in this study to further investigate the potential of lyophilization as a method to maintain the stability and functionality of EVs at ambient temperatures. This approach addresses a major limitation for the large-scale production and application of EVs for therapeutic purposes, which typically require continuous cold storage (−20 to −80 °C), which complicates distribution and increases the costs of transportation of EVs. Lyophilization offers a practical alternative for long-term preservation while maintaining EV bioactivity [21].

Excessive OS can also impair cellular migration by damaging key cellular components such as the cytoskeleton, membranes, and mitochondria [22]. Therefore, we investigated the effects of mEVs on enhancing cell migration, as an indicator of their potential role in supporting the recovery of BOECs compromised by stress.

Finally, to further elucidate the mechanisms underlying mEV activity, we assessed whether their effects are mediated by surface-associated components, such as external RNAs or proteins, using enzymatic treatments that selectively degrade these elements. In addition, we investigated the potential role of internal cargo by conducting in silico pathway analysis of the commonly detected mEV-associated miRNAs. We then performed LC-MS/MS based profiling of the mEV protein cargo, followed by GO enrichment analysis. Together, these approaches provide a framework for understanding how distinct elements of the mEV cargo influence BOECs function under stress.

## 2. Materials and Methods

### 2.1. Bovine Oviductal Epithelial Cell Cultures

The BOECs were cultured following the method described by Reshi et al. [23]. Briefly, oviducts, along with attached ovaries, were collected from the slaughterhouse (Rakvere, Estonia) and transported to the laboratory in saline solution at 37 °C, within 4 h of slaughter. Cell isolation was performed using an established protocol [23]. The isolated cells confirmed to express epithelial cell markers were cultured further. In each experiment, a batch of BOECs was grown in a 100 mm Petri dish using Dulbecco’s Modified Eagles medium F12 (DMEM 12-604F, Lonza, Verviers, Belgium) media supplemented with 10% Fetal Bovine Serum (FBS) (Gibco™, 10500064, Paisley, UK), 1 μL/mL amphotericin B, and 10 μL/mL penicillin/streptomycin until they reached approximately 80% confluency. To mimic the luteal phase, 100 ng/mL progesterone (P4) and 75 pg/mL estradiol 17β (E2) were added to the complete media [24].

### 2.2. Pre-Processing of Milk

The milk pre-processing and EV enrichment procedures were described previously by Sapugahawatte et al. [25]. Briefly, the source material for the production of mEVs was commercially available pasteurized low-fat milk (Tere joogipiim, TERE AS, Lelle 22, Tallinn 11318, Estonia) (*n* = 3). The milk curdled when the pH was lowered to 4.6 using 0.5% glacial acetic acid (Thermo Fisher Scientific, Waltham, MA, USA) and the whey liquid separated. Milk was allowed to curdle at +4 °C for one hour. The curds were then separated using Whatman grade 4 qualitative filter papers (Cytiva, Marlborough, MA, USA) and 0.45 µm bottle top dead-end filters (Corning®, Sigma Aldrich, Burlington, MA, USA).

### 2.3. Pre-Processing of Spent Algae Culture Media

Chlorella vulgaris 211-11b cultures were grown in a photobioreactor under CO_2_-enriched conditions, with continuous aeration and a 14/10 light/dark photoperiod using direct illumination, and maintained until the late-logarithmic growth phase. The culture media from the algae was used for EV enrichment. Initially, it was gravity-filtered through grade 292 filter paper (5–8 µm pore size). The filtrate was then collected for further processing using tangential flow filtration (TFF).

### 2.4. EV Enrichment Process

A tabletop TFF system (Centramate, Cytiva, MA, USA) with a 300 kDa molecular weight cutoff polyethersulfone membrane filter (Omega PES membrane, 0.02 m^2^ surface area, Cytiva, MA, USA) operating in a closed circle was used to concentrate the collected filtrates from the previous step. TFF was continued until the necessary enrichment was obtained. Buffer exchange was performed with Dulbecco’s PBS (Sigma-Aldrich Co., St. Louis, MO, USA). Finally, the concentrated samples were stored at −80 °C following a 3000× *g* centrifugation.

### 2.5. Lyophilizing Procedure

EV samples (120 µL at 10^12^ particles/mL), frozen at −80 °C, were subjected to a freeze dryer (Christ, Alpha 2-4 LDplus, Osterode am Harz, Germany) with an ice condenser set at −87.8 °C and 1.3 mbar vacuum pressure. Following 24 h of freeze-drying process, lyophilized EVs were kept in 4 °C till used for experiments. On the day of experiments, 120 µL PBS was used to reconstitute the lyophilized EV sample for further use.

### 2.6. Nanoparticle Tracking Analysis

The size and concentration of the isolated EVs were determined using a ZetaView PMX nanoparticle tracking analysis (NTA) instrument (Particle Metrix GmbH, Inning am Ammersee, Germany). Instrument auto-alignment was carried out using 100 nm polystyrene bead standards (Applied Microspheres B.V., Leusden, The Netherlands; catalog no. 10100). Following alignment, all samples were analyzed in scatter mode to determine particle size distribution and total particle counts. Measurements were acquired using a camera sensitivity of 85, a shutter value of 70, and a frame rate of 30 frames per second (fps), with three acquisition cycles consisting of 11 frames each. Data were collected from three independent biological replicates.

### 2.7. Transmission Electron Microscopy

Morphological assessment of EVs was performed using transmission electron microscopy (TEM), following the general procedure. Briefly, 20 µL of the EV preparation was applied onto formvar/carbon-coated 200-mesh grids (Agar Scientific, Stansted, UK) and left for 20 min to allow vesicle adsorption. The grids were then stained with 2% uranyl acetate (Polysciences, Warrington, PA, USA) for 5 min, air-dried, and subsequently examined using a JEM-1400 TEM (JEOL Ltd., Tokyo, Japan) equipped with a Morada CCD camera (Olympus, Hamburg, Germany) operating at 80 kV. Images were recorded digitally using the Morada camera system.

### 2.8. Sample Preparation for LC-MS/MS

Protein samples were processed in a randomized order. Proteins were precipitated using 4 mg/mL sodium deoxycholate in 100% TCA to a final concentration of 20% (*v*/*v*), incubated at 4 °C for 30 min, and centrifuged at 17,000× *g* for 15 min. Pellets were washed with acetone and methanol, air-dried, and quantified by Micro BCA assay. Dried pellets were solubilized in 7 M urea, 2 M thiourea, 100 mM ABC, and 20 mM methylamine, reduced with 10 mM DTT at 30 °C for 30 min, and alkylated with 30 mM chloroacetamide for 30 min in the dark. Proteins were pre-digested with Lys-C (1:100) for 2 h at 25 °C, diluted five-fold with 100 mM ABC, and digested overnight at 25 °C with dimethylated trypsin (1:50). Peptides were acidified to 1% TFA, purified using in-house C18 SPE tips, and resuspended in 0.5% TFA for nano-LC-MS/MS analysis.

### 2.9. LC-MS/MS Analysis

LC-MS/MS analysis was performed using an Ultimate 3000 RSLCnano system (Dionex, California, USA), where injected peptides were first loaded onto a 0.3 × 5 mm trap-column (5 µm C18 particles, Dionex). The peptides were then transferred to an in-house packed (3 µm C18 particles, Dr Maisch, Ammerbuch, Germany) analytical 50 cm × 75 µm emitter-column(MS Wil, CoAnn Technologies, Aarle-Rixtel, The Netherlands) and separated using a 60 min gradient from 8% to 40% of buffer B (buffer A: 0.1% formic acid, buffer B: 80% acetonitrile + 0.1% formic acid). The separated peptides were subsequently ionized via nano-electrospray and introduced into a Q Exactive HF mass spectrometer (Thermo Fisher Scientific) operating in positive mode with a spray voltage of 2.5 kV. Eluted peptides were ionized by nano-electrospray (2.5 kV) and analyzed on a Q Exactive HF mass spectrometer in data-dependent acquisition mode (top-12). Full MS scans were acquired from *m*/*z* 350–1400 at 60,000 resolution, followed by HCD MS/MS at 30,000 resolution using NCE 26, a 1.6 *m*/*z* isolation window, and dynamic exclusion of 30 s. Target values were 3,000,000 ions for MS and 100,000 ions for MS/MS with maximum injection times of 50 ms and 45 ms, respectively.

### 2.10. Raw Data Analysis

LC-MS/MS data were processed with MaxQuant (v2.2.0.0). Methionine oxidation and N-terminal acetylation were set as variable modifications, and cysteine carbamidomethylation as a fixed modification. Spectral searches were performed against the Bos taurus UniProt proteome with trypsin specificity and up to two missed cleavages, including a contaminants database but excluding bovine serum proteins. Only identifications with at least 1 peptide ≥ 7 amino acids long (with up to 2 missed cleavages) were accepted, and transfer of identifications between runs based on accurate mass and retention time was enabled. Label-free normalization with MaxQuant LFQ algorithm was also applied. LFQ ratio count (i.e., number of quantified peptides for reporting a protein intensity) was set to 2. Peptide-spectrum match and protein false discovery rate (FDR) was kept below 1% using a target-decoy approach. All other parameters were default.

### 2.11. Bioinformatics Analysis for Protein Expression Comparison

Differential expression analysis was performed in R (v4.2.1) using the DEP package (version 1.28.0). Contaminant and reverse-sequence proteins were removed, and only proteins with at least two valid values in one group were retained. Data were normalized using variance stabilizing normalization, and missing values were imputed assuming low signal intensity. Protein-specific linear models with empirical Bayes moderation were applied, and *p*-values were corrected using the Benjamini–Hochberg FDR method. Proteins with an adjusted *p* < 0.05 and log2 fold change > 1 between lyophilized EVs versus frozen EVs were considered significantly differentially expressed.

### 2.12. Preparation of EV-Depleted Medium

EV-depleted FBS was prepared using the previously established protocol [26]. FBS was filtered at 3000× *g* for 55 min using Amicon Ultra15 centrifugal filters (100 kDa, Merck KGAA, Darmstadt, Germany) as previously described [26]. Filtered FBS was added as a 10% supplement to prepare complete culture media for BOEC treatment with EVs.

### 2.13. Viability/Cytotoxicity Cell Analysis Utilizing Resazurin Assay

Cell viability was checked using 50 µmol/L resazurin solution prepared in PBS containing Ca^2+^ and Mg^2+^ according to the previously published protocols [27]. Flat-bottom 96-well plates with a density of 10,000 cells/well were used for this assay. After the treatment period, the cells were washed and the media was replaced by the resazurin solution. The plate was placed in a multimode reader Cytation 5 (Biotek, Winooski, VT, USA), and the absorbance was measured at 570 nm and 600 nm using a monochromator in kinetic mode, with readings taken every 15 min for 2 h at a read height of 8.5 mm and with the lid on. The absorbance ratio at two different wavelengths was used for the analysis.

### 2.14. Cell Migration Assay

A migration assay in the presence of an anti-proliferative drug mitomycin C (SIGMA-ALDRICH) was performed to assess the impact of EVs on cell migration. Before assessing migration, we first verified that mitomycin C effectively inhibited proliferation. BOECs were cultured in a 24-well plate until a full confluency monolayer was formed. The cells were pre-treated with mitomycin C for 2 h. Then, the cell numbers were quantified using trypan blue immediately after scratching (0 h) using a 200 μL pipette tip, and again after 24 h. This confirmed that wound closure in the subsequent assay reflected motility rather than proliferation.

For the migration assay, similarly, BOECs were cultured in a 24-well plate until a full confluency monolayer was formed. BOECs were pre-treated with 10 µg/mL mitomycin C for 2 h to suppress proliferation, ensuring that the assay specifically measured cell migration. Artificial separation lines were created in the monolayer using a 200 μL pipette tip. After scratching, cells were washed and incubated in an EV-depleted medium containing EVs at concentrations of 10^9^ EVs/mL (*n* = 3). Control cells were maintained in the same medium without treatment.

Phase contrast images were captured at 0, 8, 16, and 24 h post-scratching using an inverted light microscope (Zeiss Axiovert 200, Göttingen, Germany) and recorded with a digital camera (Axiocam 202 mono, Göttingen, Germany). To ensure consistent imaging of the same area over time, reference marks were made on the bottom of each well [28].

The scratched area was measured at each time point using the ImageJ software version 1.54p with the wound-healing-size-tool plugin. The percentage of cell migration was calculated relative to the initial wound area at 0 h using the following formula:

$$\mathrm{Cell}\;\mathrm{migration}\;(\%)\;=\;\frac{Cell\;free\;area\;at\;0\;h\;-\;Cell\;free\;area\;at\;time\;t}{Cell\;free\;area\;at\;0\;h}\times 100$$

### 2.15. Surface Modification of mEVs

Protease and RNase treatments of mEVs were carried out based on a previously established protocol [29] with slight modifications. 30 µL of reconstituted lyophilized mEVs was treated with 2 µg/mL proteinase K (PK) (Thermo Scientific, REF EO0491, Waltham, MA, USA) for 30 min at 37 °C. This was followed by the addition of 5 mM phenylmethylsulfonyl fluoride (PMSF) to inhibit protease activity, incubating for 10 min at room temperature. RNase A (RA) treatment was performed by adding 8 µg/mL RNase A (Thermo Scientific, REF EN0531) to the mixture and incubated at 37 °C for 15 min under gentle agitation. Afterward, RNase inhibitor (cat. no EO0381, ThermoFisher, Waltham, MA, USA) with the concentration of 1 U/μL was added to the sample for 5 min at room temperature.

EV detergent treatment (positive control) was carried out by adding Triton X-100 (TX) (Bio-Rad, Cat. No. 1610407) to 30 µL of reconstituted lyophilized mEVs to reach a final concentration of 0.1% (*v*/*v*) and vortex for 30 s at room temperature.

The EV samples (protease and RNase treated and positive controls) were diluted with PBS to a final volume of 100 µL and washed using Exosome Spin Columns (MW 3000, Invitrogen (Carlsbad, CA, USA), REF 4484449) following the manufacturer’s protocol to remove residual treatments [30].

Protein quantification before and after treatments for Protease and RNase treated EVs and positive controls was carried out using the Bradford assay, following previously established protocols [31]. In brief, a series of dilutions of bovine serum albumin (BSA) standard solution (2 mg/mL, Sigma-Aldrich, AU: St Louis, MO, USA) was prepared to generate a standard curve with concentrations ranging from 1.4 to 0.125 mg/mL. PBS used as the negative control. Absorbance readings for both the standards and samples were obtained using a spectrophotometer (Ledetect 96 Microplate Reader; Biomed Dr. Wieser GmbH, Salzburg, Austria) at a wavelength of 620 nm, and protein concentrations for each sample were subsequently determined.

To ensure effective surface modification, RNA quantification and quality control were performed on EV samples (Protease and RNase treated and positive controls). Total RNA was isolated from 100 µL of EVs using the Qiagen miRNeasy Mini Kit (Germantown, MD, USA) according to the manufacturer’s protocol. The concentration and integrity of the extracted RNA before and after RNase treatment were assessed using a Bioanalyzer Automated Electrophoresis system (Agilent Technologies, Santa Clara, CA, USA) with the Agilent RNA 6000 Pico Kit (Agilent Technologies, cat. No. 5067–1513) [32].

### 2.16. RNA Extraction and RNA Quality Control from BOECs

Total RNA was extracted from the BOECs using TRIzol Reagent (Invitrogen). Two microliters of UltraPureTM Glycogen (Cat. no. 10814–010, Thermo Fisher Scientific, Bleiswijk, Netherlands) was added to each sample’s lysis solution to improve the efficiency of RNA extraction. To ensure purity, the RNA precipitate was washed with 70% ethanol three sequential times. Nanodrop 2000 spectrophotometer (Thermo Scientific, Wilmington, DE, USA) was used to measure the absorbance of the RNA samples at 260 nm for the quantitative and qualitative analysis.

### 2.17. Quantitative Real-Time PCR

The mixture of random primers, oligo(dT) and FIREScript RT cDNA synthesis kit (Solis BioDyne, Tartu, Estonia) was used for reverse transcription of RNA. Using the EvaGreen assay system (Solis BioDyne, Tartu, Estonia), cDNA products were amplified in triplicate for every biological sample. An initial denaturation stage at 95 °C for 15 min was followed by 40 cycles of 95 °C for 20 s, 62 °C for 20 s, and 72 °C for 20 s as part of the amplification program. The comparative CT method was used for the qPCR data analysis, and the 2-ΔΔCT method was used to determine the relative RNA expression [33]. The relative expression of DNA damage inducible transcript 4 (*DDIT4)* and Hypoxia-Inducible Factor 1-alpha (*HIF1A)* was investigated as the target genes. The genes beta-2-microglobulin (*B2M*) and TATA-binding protein (*TBP*) were utilized for normalization. Using Primer-BLAST, primers (Table 1) were created, favoring sequences that cross exon-exon junctions for increased specificity.

### 2.18. Prediction of Potential Targets of Common miRNAs in mEVs

The list of most abundant mEV miRNAs, which were highly conserved between mammals [34], was acquired from a previous report by Saenz-de-Juano et al. (Gene Expression Omnibus accession code GSE232617) [35]. TargetScan database (version: release 8.0) was used to identify the mRNA targets of each miRNA. Target mRNA with Cumulative Weighted Context++ score < −0.5 were selected for further analysis. Since the TargetScan database provides human ortholog of target genes, BLAST tool (version 2.14.1) was used to align transcript sequences between human and cattle genomes. Afterwards, cattle homologs of all the genes with pident > 70 and bitscore > 50 were selected. The results were merged with the output of TargetScan database based on ENSEMBL IDs, therefore the potential mRNA targets in cattle which can be influenced by miRNA were detected.

### 2.19. Gene Ontology (GO) Enrichment Analysis of Predicted Targets of mEVs miRNA

GO enrichment analysis was performed to identify overrepresented pathways associated with the gene set of interest. Ensembl gene annotations for *Bos taurus* were obtained using the biomaRt R package (version 4.4.2) [36]. The required datasets were retrieved from the Ensembl genome database. Enriched pathways were visualized using dot plots representing fold enrichment, statistical significance, and gene counts for each pathway. Pathway descriptions were ordered by fold enrichment values to facilitate interpretation.

### 2.20. Functional Annotation and Pathway Enrichment Analysis of mEV Proteins

Functional annotation and enrichment analysis were performed in R using clusterProfiler (version 4.14.6). Protein identifiers, without significant changes between the groups, were first converted from UniProt to Entrez IDs using the bitr function (org.Bt.eg.db) (version 3.22.0), and duplicate Entrez IDs were removed. Gene Ontology (GO) enrichment was then conducted with enrichGO using the Bos taurus annotation database, evaluating all GO domains (BP, CC, MF). Enrichment results were adjusted for multiple testing using the Benjamini–Hochberg method, applying significance thresholds of *p*-adjusted < 0.05 and q-value < 0.2. This approach enabled the identification of significantly enriched biological processes, cellular components, and molecular functions based on the submitted protein list.

### 2.21. Statistical Analyses

All experiments were conducted in triplicate. The normality of the data distribution was verified using the Kolmogorov–Smirnov test. Differences in cell viability between different groups were analyzed using two-way ANOVA performed with GraphPad Prism 7 software (GraphPad Software Inc., San Diego, CA, USA). Except proteomic analysis, for which four samples per group were analyzed, all experiments were conducted using three independent biological replicates, each measured with three technical replicates to ensure analytical rigor and reproducibility. Results are presented as the mean ± standard deviation (SD). Statistical significance was considered at *p* < 0.05.

### 2.22. Experimental Design

#### 2.22.1. Does Lyophilization Affect the mEVs Characteristics?

Particle size and concentration of mEVs before and after lyophilization were measured using the NTA instrument for 3 biological replicates per group. Three technical measurements were performed for each sample. Furthermore, the morphology of mEVs was checked by transmission electron microscopy (TEM). The comparative proteomic analysis of lyophilized and frozen mEVs was conducted using label-free quantitative proteomics.

#### 2.22.2. What Is the Minimum Concentration of CoCl_2_ Needed to Reduce the Cell Viability?

Experiments were conducted to determine the minimum concentration of CoCl_2_ required to induce cell death following 24 h of CoCl_2_ exposure. BOECs were seeded at a density of 10^4^ cells per well in 96-well plates and cultured overnight. When the cells reached 70–80% confluency, they were treated with 100, 200, 400, and 600 µM CoCl_2_ for 24 h. Cytotoxicity of CoCl_2_ at each concentration was assessed using the resazurin assay. Experiments were performed in triplicate in three separate days.

#### 2.22.3. Can EVs Induce Cytotoxic Effects on Cells?

To assess the impact of EVs on cell viability, BOECs seeded at a density of 10^4^ cells per well in 96-well plates and cultured overnight to reach 70–80% confluency. Then the cells were treated with EVs stored in frozen and lyophilized form with different concentrations of mEVs (10^7^, 10^9^, and 10^11^ EVs/mL) and aEVs (10^7^, 10^9^, and 10^11^ EVs/mL), respectively, for 24 h. EV cytotoxicity was assessed using the resazurin assay. Experiments were performed in triplicate on three separate days.

#### 2.22.4. Can mEVs Prevent or Overcome CoCl_2_-Induced OS in BOECs?

To investigate the potential of mEVs and aEVs to suppress the cytotoxic impact of CoCl_2_, three experimental conditions were applied: (i) EV pre-treatment group: this group of BOECs were pretreated for 4 h with EVs, followed by 24 h of CoCl_2_ exposure; (ii) EV co-treatment group: In this group BOECs was co-incubated with EVs and CoCl_2_ for 24 h and; (iii) EV post-treatment group; in this group after 24 h CoCl_2_ treatment of BOECs, cells were incubated for 4 h with different EVs. Thereafter, cell viability/cytotoxicity was assessed utilizing resazurin assay. In this experiment, 200 µM CoCl_2_ was used with (10^7^, 10^9^, and 10^11^ EVs/mL) mEVs and aEVs. In addition, EVs that were stored frozen and lyophilized were tested in triplicate on three different experimental days.

#### 2.22.5. Can EVs Influence BOECs Cell Migration?

Cell count analysis was performed to confirm that mitomycin C effectively inhibited proliferation. Then, the impact of EVs on cell migration was investigated. Lyophilized EVs with a concentration of 10^9^ EVs/mL were added to the cells following scratching, and the cells were incubated for 24 h. Microscopic images with 8 h intervals were obtained to evaluate the impact of EVs on cell migration. This experiment was conducted in triplicate on three independent days.

#### 2.22.6. Is the Preventive/Recovery Effect of mEVs Dependent on EV Surface Molecules?

To investigate whether the protective effects of mEVs against CoCl_2_-induced OS in BOECs was dependent on EV surface molecules, BOECs were co-treated with 200 µM CoCl_2_ and with either (i) non-treated intact mEVs or (ii) PK treated mEVs or (iii) PK and RA treated mEVs or (iv) TX treated mEVs (10^9^ EVs/mL) for 24 h. To determine whether surface-modified mEVs retained their protective effects, cell viability was assessed using the resazurin assay in 96-well plates with a density of 10,000 cells/well. In parallel, the molecular impact of mEVs treatment was examined by quantifying the expression of DDIT4, a gene known to be upregulated under oxidative stress. For gene expression analysis, BOECs were seeded in 24-well plates (10^5^ cells/well) and co-incubated with CoCl_2_ and 10^9^ EVs/mL for 24 h in all the 4 groups (as mentioned elsewhere). Following treatment, total RNA was extracted using 250 µL of TRIzol reagent, and DDIT4 expression was analyzed via real-time qPCR. The experiment was performed for 3 biological replicates accompanied by 3 technical measurements.

#### 2.22.7. What KEGG Pathways in BOECs Are Affected by mEV miRNA?

We hypothesized that mEVs miRNA affects signaling pathways which can mediate the BOEC stress response and cell migration. To test this hypothesis, an in silico analysis was performed using the most abundant and conserved mEV miRNAs reported by Saenz-de-Juano et al. [34]. Predicted mRNA targets were identified using the TargetScan database (v8.0) and filtered based on context scores. Human targets were then mapped to their bovine homologs using BLAST, allowing the identification of potential bovine gene targets involved in cellular stress regulation. Furthermore, to explore the potential pathways involved in the miRNA-mediated response to oxidative stress, GO enrichment analysis was performed on the predicted target genes.

#### 2.22.8. Which Functional Pathways Are Enriched in the mEV Proteome?

We hypothesized that mEV-associated proteins may modulate signaling pathways governing the BOEC stress response and cell migration. To investigate this, GO enrichment analysis was subsequently performed on all the identified proteins in frozen and lyophilized mEVs to elucidate functional pathways potentially contributing to the protein-mediated response under oxidative stress.

## 3. Results

### 3.1. Comparison of Particle Size, Concentration, and Morphology of Frozen and Lyophilized mEVs

**Figure 1:**
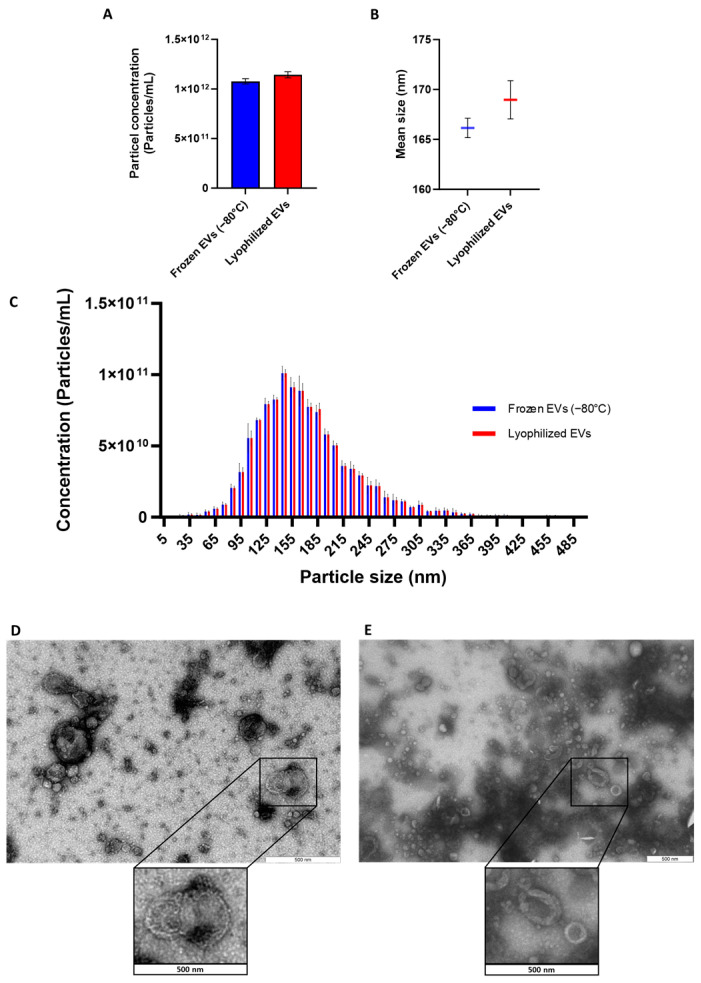
Concentration, size, and morphology of frozen and lyophilized milk EVs. No significant differences were detected in (**A**) particle concentration, (**B**) average size, and (**C**) size distribution of mEVs. Values are presented as mean ± SD, of 3 biological replicates (*n* = 3) accompanied by 3 technical replicates. TEM images show similar morphology of (**D**) Frozen mEVs and (**E**) Lyophilized mEVs.

The NTA analysis demonstrated that there were no significant differences in the concentration and size of the particles between lyophilized and frozen groups (Figure 1A,B). Size distribution analysis demonstrated a similar pattern for lyophilized and frozen mEVs (Figure 1C). Moreover, TEM analysis depicts a similar morphology of EVs before and after lyophilization (Figure 1D,E). Enrichment of EV surface markers in frozen and lyophilized mEVs was confirmed by Western blotting and reported previously [25].

### 3.2. Comprehensive Proteomic Profiling Shows Overall Stability of the mEV Proteome After Lyophilization

**Figure 2:**
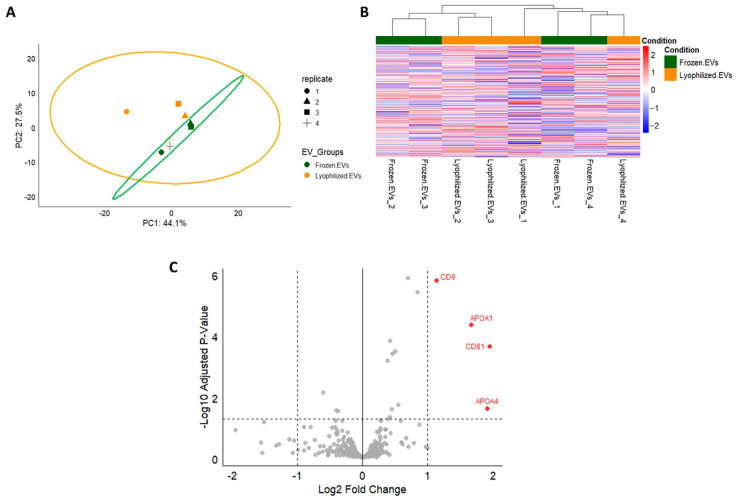
Proteomic comparison of lyophilized versus frozen mEVs. (**A**) Principal component analysis (PCA) plot showing substantial overlap of 95% confidence regions, indicating similar variance structure across groups. Frozen and lyophilized EVs marked in green and orange, respectively. (**B**) Heatmap displaying no distinct clustering, consistent with minimal group separation. (**C**) Volcano plot identifying four proteins significantly enriched after lyophilization (log2FC > 1; BH-adjusted *p*-values < 0.05). The analysis was performed with 4 biological replicates per group.

The comparative proteomic analysis demonstrated high similarity between lyophilized and frozen mEVs. Principal component analysis (PCA) and heatmap revealed substantial overlap among groups, with no distinct clustering, and the volcano plot identified only a small number of significantly altered proteins (Figure 2).

### 3.3. CoCl_2_ and EV Effect on BOEC Viability/Cytotoxicity

**Figure 3:**
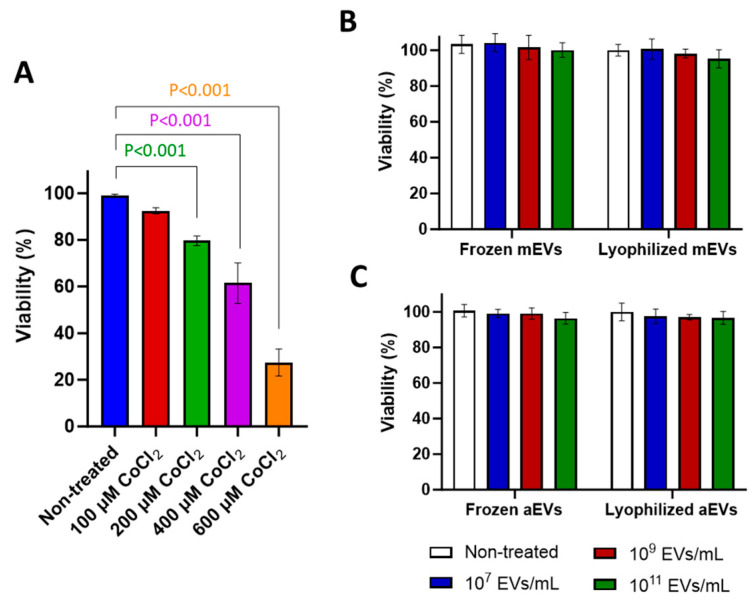
Effect of CoCl_2_, milk EVs (mEVs) and algae EVs (aEVs) on BOECs cell viability. BOECs viability after treating with (**A**) CoCl_2_, (**B**) mEVs, and (**C**) aEVs. Values are presented as mean ± SD, of 3 biological replicates (*n* = 3) with 3 technical measurements. *p* < 0.05 was considered as statistically significant.

The minimum dose of CoCl_2_ required to significantly (*p* < 0.001) reduce cell viability was 200 µM of CoCl_2_ (Figure 3A). Different concentrations of mEVs and aEVs stored either in frozen or lyophilized forms had no significant impact on cell viability (Figure 3B,C).

### 3.4. mEVs, Unlike aEVs, Significantly Reduced the Toxicity Effect of CoCl_2_ on BOECs in All Treatment Groups

**Figure 4:**
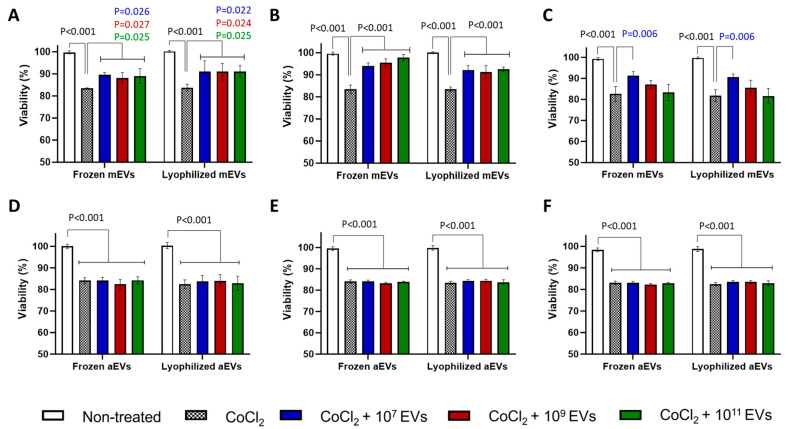
Effects of milk and algae EVs (mEVs and aEVs) on BOEC viability under CoCl_2_-induced stress. Viability of BOECs treated with mEVs under three experimental conditions: (**A**) mEVs pre-treatment, (**B**) mEVs co-incubation, (**C**) mEVs post-treatment. Corresponding viability analyses using aEVs: (**D**) aEVs pre-treatment, (**E**) aEVs co-incubation, (**F**) aEVs post-treatment. Values are presented as mean ± SD of 3 biological replicates (*n* = 3) with 3 technical measurements. *p* < 0.05 was considered as statistically significant.

Presence of mEVs at varying concentrations in EV Pre-Treatment and EV Co-Treatment groups led to a significant increase in cell viability in all groups (Figure 4A–C). In EV Post-Treatment group, a significant recovery effect (*p* = 0.006) was observed only at a concentration of 10^7^ mEVs particles/mL (Figure 4C). On the other hand, the presence of aEVs had no significant impact on cell viability compared to CoCl_2_-treated cells, in any of the treatment groups (Figure 4D–F). Notably, EVs stored either frozen at −80 °C or lyophilized exhibited comparable effects on cell viability across all experimental conditions.

### 3.5. Milk EVs (mEVs) Outperformed Algae EVs (aEVs) in Promoting Cell Migration

**Figure 5:**
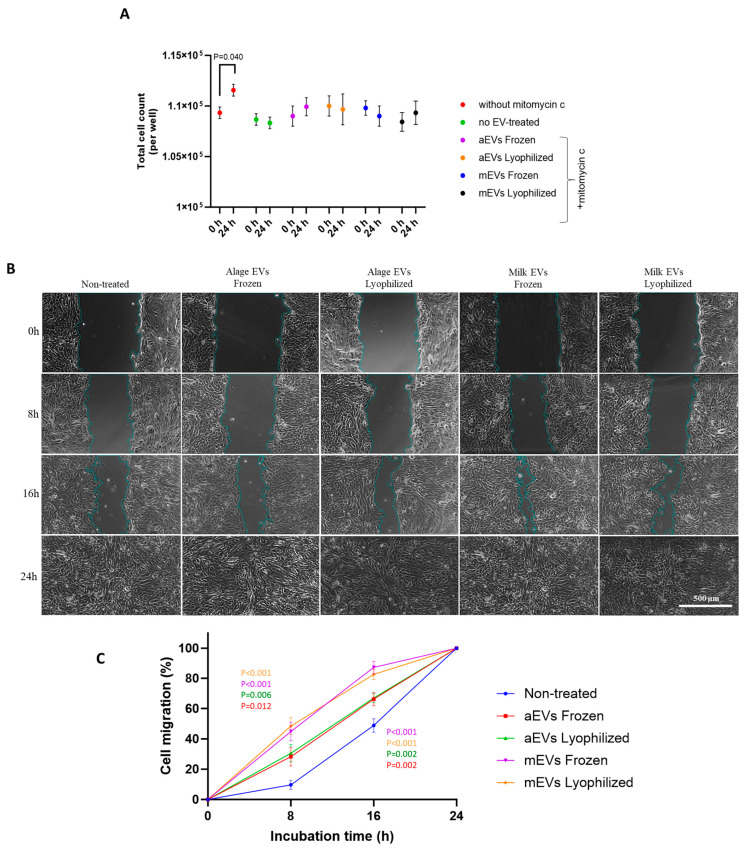
Assessment of mitomycin C efficacy and the impact of milk EVs (mEVs) and algae EVs (aEVs) on BOEC cell migration. (**A**) Mitomycin C effectively inhibited BOEC proliferation, as indicated by stable cell numbers during the assay, while untreated cells showed a significant rise in cell count. (**B**) Representative microscopic images showing BOECs cell migration at 0, 8, 16 and 24 h after treatment with mEVs and aEVs by the migration assay. (**C**) Quantitative analysis of cell migration rate. Values are presented as mean ± SD of 3 biological replicates (*n* = 3) with 3 technical measurements. *p* < 0.05 was considered statistically significant.

Mitomycin C treatment effectively suppressed cell proliferation, as shown by the absence of a significant increase in cell number during the EV-treatment period, whereas a marked proliferative rise was observed in the group without mitomycin (Figure 5A).

Microscopic images in Figure 5B illustrate enhanced cell migration by different EV treatments, evidenced by reduced scratch area at later time points compared to the initial stage. Both mEVs and aEVs significantly enhanced BOECs migration in different time points. However, the effect was more pronounced in cells treated with mEVs, as indicated by a higher percentage of cell migration compared to aEV treatments (Figure 5C). Moreover, EVs stored as lyophilized or frozen form induced similar impact on cell migration.

### 3.6. Surface-Modified mEVs Retain Their Potential to Reduce the Cytotoxic Effect of CoCl_2_

**Figure 6:**
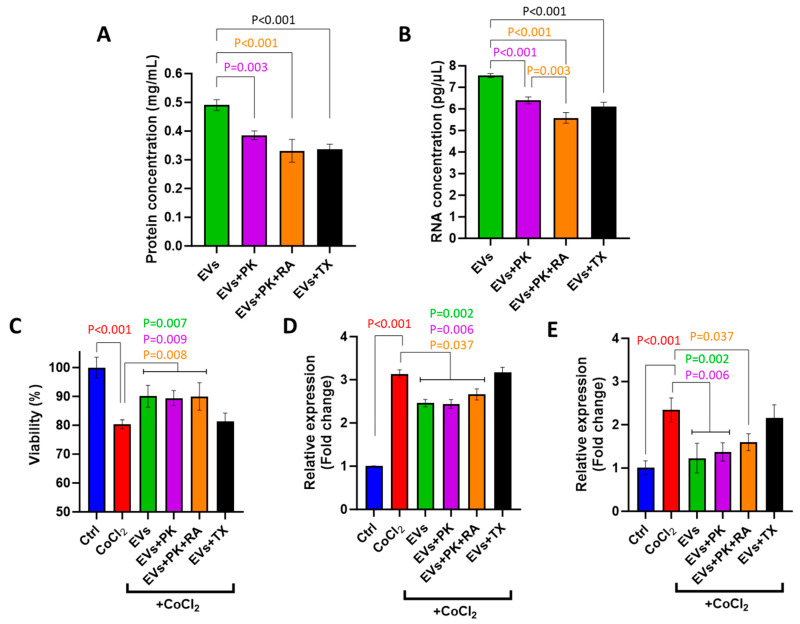
Changes in protein and RNA concentration of surface-modified mEVs by proteinase K treatment (PK), RNA A treatment (RA), mixture of PK and RA treatment, and Triton X100 (TX) treatment (**A**,**B**), and their impact on BOEC viability (**C**), *DDIT4* and *HIF1A* expression (**D**,**E**). Values are presented as mean ± SD of 3 biological replicates (*n* = 3) with 3 technical measurements. *p* < 0.05 was considered statistically significant.

Concentrations of surface proteins and RNA were significantly reduced in PK-treated mEVs as well as PK- and RA-treated mEVs and TX-treated mEVs compared to non-treated intact mEVs (Figure 6A,B).

The surface modification of mEVs did not alter their recovery potential, as they maintained their ability to improve cell viability (Figure 6C) and suppress the expression of *DDIT4* and *HIF1A* (Figure 6D,E).

### 3.7. Pathway Analysis of Predicted Targets of Common miRNAs in mEVs

**Figure 7:**
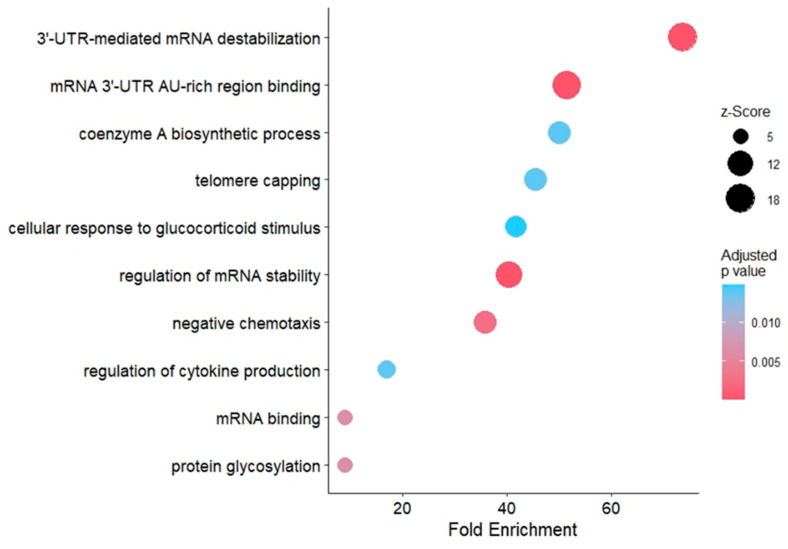
GO term analysis of predicted targets of common miRNA in mEVs. GO analysis revealed the significant enrichment of pathways related to cytokine production, glucocorticoid response, and negative chemotaxis, alongside other general molecular processes. The color and size of the circle points indicate the adjusted *p*-value and z-score, respectively. Pathways were arranged according to their fold enrichment score.

GO term analysis revealed enriched pathways in general molecular processes such as mRNA binding, regulation of mRNA stability and negative chemotaxis, as well as more specific pathways including regulation of cytokine production and cellular response to glucocorticoid stimulus (Figure 7). These exploratory findings may indicate mechanisms through which mEVs contribute to cellular responses under OS conditions, and they provide a basis for future validation studies. A summary of the major pathways and their functional implications according to previous literature is provided in Table 2.

### 3.8. Pathway Analysis of Frozen and Lyophilized mEV Proteins

**Figure 8:**
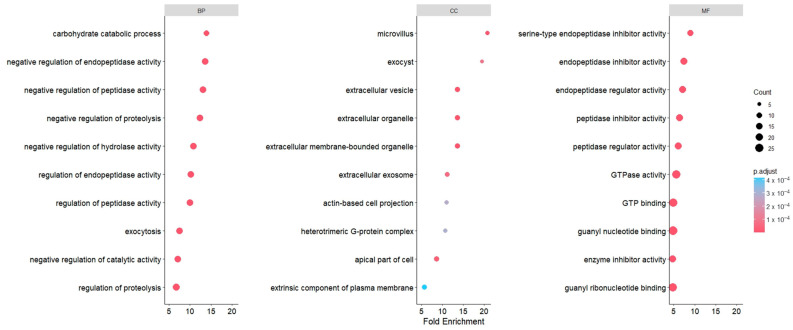
GO term analysis showed enrichment of peptidase- and endopeptidase-related pathways, along with cellular component terms associated with extracellular vesicles, exosomes, organelles, and exocyst-related processes. The color and size of the circle points indicate the adjusted *p*-value and protein count, respectively. Pathways were arranged according to their fold enrichment score.

GO term analysis indicated that the most enriched pathways were associated with peptidase and endopeptidase functions, appearing across both the biological process and molecular function categories. In the cellular component domain, enriched terms were predominantly linked to extracellular vesicles, exosomes, organelles, and exocyst pathways (Figure 8). Together, these results highlight molecular processes that could be relevant to mEV activity under oxidative stress and provide direction for future mechanistic validation studies.

## 4. Discussion

OS can disrupt cellular homeostasis in reproductive tissues, where maintaining redox balance is essential. OS poses a significant threat, particularly to oviductal epithelial integrity, where tight regulation of redox homeostasis is critical for supporting gamete transport, fertilization, and early embryo development [43]. Excessive production of ROS in the oviductal environment, often triggered by inflammation, infection, or metabolic imbalance, can disrupt cellular metabolism, impair epithelial function, and compromise reproductive success [44,45]. Given the detrimental effects of OS on oviductal physiology, it is essential to identify strategies that can mitigate its impact and restore epithelial cell viability. This study was designed to evaluate the effects of mEVs on BOECs recovery when administered before, during, or after CoCl_2_-induced OS. Notably, the MISEV2018 guidelines for functional studies of EVs recommend the inclusion of EVs from different sources as negative controls to evaluate potential non-specific effects [46]. Accordingly, EVs derived from the spent culture medium of a specific algae (Chlorella vulgaris) were used in this study. To assess the protective potential of mEVs against OS, we employed a resazurin-based viability assay, which is widely recognized as a sensitive method for monitoring metabolic activity in various cell types [27,47], making it suitable for assessing the protective effects of EVs on OS conditions.

In the current investigation, BOECs treated with CoCl_2_ alone exhibited significantly reduced viability with the minimum concentration of 200 µM, confirming its cytotoxic effects. This finding aligns with previous reports demonstrating that CoCl_2_ impairs cell viability by increasing ROS levels and triggering apoptosis in multiple cell types [20,48]. CoCl_2_, as an indirect inducer of hypoxia-inducible factors (*HIFs*), has been used in in vitro studies to simulate physiological OS conditions, thereby triggering cellular responses similar to those observed under oxygen-deprived conditions [49,50,51].

Further experiments were performed to make sure that the concentrations of EVs used in our study were not cytotoxic to BOECs. Interestingly, our findings revealed that even at high concentrations, mEVs and aEVs did not cause cytotoxicity. Similarly, it has been reported that mEVs had no cytotoxicity in RAW264.7 cells even at high concentrations, and no systemic toxicity was observed in mice following the highest tested dose (150 µg/injection) [52]. In any experimental system, it is critical to confirm that the effects observed upon exposure to different molecules or biological materials are attributable to specific biological activity rather than nonspecific cytotoxicity. In such contexts, careful selection of the EV source and optimization of dosage are essential to avoid unintended cellular damage and to ensure biocompatibility [53]. This result not only strengthens the reliability of our experimental outcomes but also supports the potential use of mEVs in therapeutic applications, as they appear to be well-tolerated by cells. This aspect of our work emphasizes the importance of dose testing in EV research and provides an encouraging perspective on the safety profile of mEVs for future biomedical use.

To ensure compatibility with larger-scale applications, we implemented a tangential flow filtration (TFF)–based enrichment strategy designed to support scalable EV processing. While the possibility of co-isolated bioactive components cannot be completely excluded, our previous characterization as well as proteomic analysis for mEVs, confirmed the presence of EV-specific protein markers including CD9 and CD81 [25], supporting that the observed biological effects in this study are largely attributable to the EV fraction. In the present study, milk was subjected to a mild acid-precipitation step prior to tangential flow filtration, which promotes aggregation and removal of casein micelles, thereby reducing protein carryover into the EV fraction. However, TFF cannot completely eliminate residual casein or other soluble milk proteins, and this represents a potential limitation of the current methodology. Further refinement of isolation techniques will be important in future research to more clearly distinguish EV-specific effects from potential contributions of co-isolated molecules.

EVs hold substantial promise for immune therapeutics and drug delivery, yet their broader clinical and industrial use is limited by the requirement for −80 °C storage, which complicates handling and distribution. Lyophilization has emerged as a practical alternative, enabling long-term, room-temperature preservation while maintaining EV structure and bioactivity. Previous work has shown that freeze-drying can effectively stabilize biopharmaceuticals, including EVs from multiple sources, when suitable protectants are used [54]. In this study, basic characterization and protein profile of the mEVs before and after lyophilization revealed no major differences between the groups. This preservation is likely attributable to water removal without disruption of membrane architecture, supporting the retention of cargo integrity and uptake capacity upon rehydration.

The functionality of mEVs was evaluated using BOECs treated with both lyophilized and frozen preparations. Our findings indicate that lyophilized mEVs retain comparable functional activity to those stored at −80 °C. mEVs consistently demonstrated a protective effect against the cytotoxic impact of CoCl_2_ across all three treatment groups, including (i) EV pre-treatment, (ii) EV co-treatment, and (iii) EV post-treatment groups. Interestingly, no differences were observed between EV concentrations in pre- and co-treatment groups. Despite testing a wide range of particle concentrations, the functional responses remained largely comparable across doses. This may be explained by the possibility that EV bioactivity reaches saturation at relatively low particle numbers. Resolving this will require finer titration within the low-dose range and assessment of shorter treatment time frames in future studies.

Our results demonstrated that mEVs in the pre-treatment group protect the BOECs against CoCl_2_-induced OS. In this regard, previous research has demonstrated that pre-treatment with mEVs can exert protective effects against OS by attenuating purine nucleotide catabolism and improving cellular energy status. Pre-treatment of IEC-6 cells with milk exosomes under OS conditions resulted in a significant shift in purine nucleotide profiles, characterized by decreased levels of GMP, AMP, and IMP, alongside increased levels of ADP and ATP [55]. Moreover, milk exosome treatment significantly reduced the activities of adenosine deaminase (ADA) and xanthine oxidase (XOD), two specific enzymes involved in purine catabolism [55]. The metabolic activity of ADA and XOD is known as a particular factor that can lead to oxidative damage and inflammatory responses [56]. ADA also regulates intermediate metabolites, such as adenine and deoxyadenosine, which are cytotoxic at high concentrations. Reduced ADA activity enhances adenosine receptor interactions, contributing to anti-inflammatory effects and tissue protection [57]. These findings suggest that mEVs protect cells by preserving energy balance and reducing oxidative by-products.

Considering the recovery impact of mEVs against OS in the co-treatment group, the protective effect may result from the rapid delivery of bioactive cargos such as miRNAs, enzymes, and lipids that modulate intracellular redox balance and apoptosis. For instance, EVs are known to carry antioxidant enzymes like superoxide dismutase (SOD) and glutathione peroxidase (GPx) with direct impact on reduction of OS [58]. Moreover, EV-associated miRNAs contribute to the suppression of OS by modulating various cellular pathways. They have been shown to downregulate NAD(P)H oxidase (NOX), reduce ROS generation, maintain mitochondrial function, and regulate ion homeostasis [59]. Additionally, EV-miRNAs can activate pro-survival signaling pathways such as PI3K/Akt and modulate antioxidant responses via the NF-κB/Nrf2 axis, ultimately enhancing cellular resilience to oxidative damage [59].

In the case of using EVs as post-treatment, they enhanced viability only at the lowest concentration tested. This outcome can be attributed to the altered cellular physiological state at the time of EV administration. Once oxidative damage has occurred, membrane integrity, metabolic activity, and uptake capacity are partially compromised, thereby restricting EV internalization and downstream functional responses [60]. In addition, the ratio of functional EV particles to the diminished population of surviving cells is reduced, and these stressed cells appear less tolerant to higher EV doses. As a result, maybe only a low EV concentration could be efficiently internalized and provide a protective effect under post-treatment conditions.

The cell-migration assay demonstrated that mEVs significantly promoted cell migration, further supporting their preserved bioactivity following lyophilization. Among the miRNAs enriched in mEVs, several have been previously reported to influence cell migration across various cell types. For instance, miR-25 mediates cell migration in different cancer cells as well as miR-21-5p, which mediates wound healing in mouse skin [61].

Overall, mEVs may exert their protective functions through multiple mechanisms, such as modulation of OS pathways, promotion of cell survival, and enhancement of cellular repair processes, making them promising candidates for mitigating oxidative damage in epithelial tissues [62,63]. Also, these findings supported the idea that mEVs can act at multiple stages of cellular damage to promote resilience and recovery under OS conditions. Moreover, using aEVs as negative controls allowed us to confirm that the observed functional effects were specific to mEVs and not attributable to generic vesicular structures or contaminants. aEVs could be enriched by TFF in the same manner as mEVs, thereby ensuring methodological comparability. Although heat-inactivated mEVs or EVs from more biologically related sources, such as blood, might provide additional insights into whether the protective effect is specific to mEVs, large-scale enrichment from blood is not practically feasible with current methods. Techniques such as size-exclusion chromatography (SEC) are widely employed to obtain high-purity EV isolates; however, their application is impractical for large-scale production [64], which is critical for translational and industrial applications of mEVs.

EVs may mitigate OS through various mechanisms such as transferring internal cargo, including proteins and miRNA, as well as surface proteins or other RNA species [65,66,67]. Previous studies have shown that the surface of small EVs can exert a protective effect against OS-induced DNA damage. This activity is thought to be mediated by antioxidant enzymes associated with the EV membrane [66]. Moreover, it has been reported that cells treated with milk-derived exosomes under OS conditions had higher levels of certain miRNAs which regulate cellular responses to the OS [67]. In our study, mEVs were subjected to treatment with RA and PK to determine whether their functional activity was linked to surface components or internal cargo. The effectiveness of these enzymatic treatments was verified by the marked reduction in both protein and RNA concentration of the EVs. Interestingly, the enzymatically treated EVs maintained their ability to protect cells from CoCl_2_-induced cytotoxicity. Based on our results, it is plausible that the functional effects of mEVs in mitigating OS may not be primarily mediated by surface-associated RNAs or proteins. While these findings suggest that surface components are unlikely to be the major contributors, they do not completely exclude a potential role for surface-mediated interactions. Thus, identification of the exact mode of action awaits further experimentation.

*DDIT4* plays a key role in cellular stress responses and is well-documented to be upregulated in response to various stimuli, particularly OS [68,69,70]. Our results showed that mEVs were able to suppress the CoCl_2_-induced upregulation of *DDIT4*, even after enzymatic treatment with only PK or a mixture of PK and RA. A similar expression pattern was observed for *HIF1A*, which also exhibited reduced induction in the presence of mEVs. Notably, previous studies have reported that indicators of oxidative stress are elevated in the blood of cows exhibiting higher *HIF1A* expression, linking this marker to systemic oxidative status [71]. It suggests that internal bioactive components, such as encapsulated miRNAs, proteins, or lipids, are delivered into the recipient cells or to the immediate microenvironment of cells to exert their functional effects. The delivery process may typically be facilitated through cellular uptake mechanisms, particularly endocytosis, micropinocytosis, or membrane fusion [72,73,74]. This could partially explain how mEVs can regulate stress-related genes like *DDIT4* and *HIF1A* even after the enzymatic removal of surface elements.

Pathway analysis of common miRNAs in mEVs revealed their involvement in several specific pathways which are potentially relevant to OS response, as summarized in Table 2. These observations suggest that mEV-associated miRNAs may contribute to mitigating OS in recipient cells by modulating different signaling pathways. Several studies have examined the uptake of milk-derived EVs and the consequent transfer of their microRNA cargo into recipient cells [75]. Although uptake analysis was not included within the scope of the present work, previous studies have shown milk EVs to be internalized by a wide range of recipient cell types, including those from different species, through both passive and active uptake mechanisms, subsequently inducing functional responses in target cells [76,77]. Regarding the protein profile comparison of lyophilized versus frozen mEVs, only a small subset of proteins (CD9, CD81, APOA1, APOA4) differed significantly between lyophilized and frozen mEVs. These proteins are commonly associated with membrane organization and the EV-associated protein corona. Such changes are consistent with minor preservation-related effects on surface-exposed components rather than broad alterations in vesicle composition. Importantly, the vast majority of proteins were maintained at comparable levels across both conditions, supporting the overall stability of the mEV proteome following lyophilization. In line with this, the enriched cellular component terms predominantly mapped to extracellular vesicles, extracellular exosomes, extracellular organelles, and exocyst-related structures, all of which are associated with EV biogenesis and vesicle trafficking. This pattern further supports the overall conservation of EV-related proteins following lyophilization.

Moreover, GO-term analysis of mEV proteins identified enriched terms related to peptidase and endopeptidase activity. Oxidative stress is known to activate proteolytic systems that remove oxidized or misfolded proteins, thereby helping to stabilize cellular homeostasis and limit the activation of stress-responsive transcriptional pathways [78]. The 20S proteasome, a major endopeptidase complex, plays a central role in this process and is selectively stimulated under ROS conditions to degrade damaged proteins in a ubiquitin-independent manner [78,79]. Because accumulated oxidized proteins can trigger or amplify stress signaling, enhanced proteolysis contributes to suppressing downstream mediators such as *HIF1A* and *DDIT4*. According to the GO-term analysis, the enrichment of peptidase and endopeptidase-related functions suggests that mEVs may support proteostasis in stressed BOECs by supplementing endogenous proteolytic capacity. Such an effect offers a biologically plausible explanation for the observed attenuation of CoCl_2_-induced *HIF1A* and *DDIT4* expression following mEV treatment, although the precise mechanisms warrant further investigation.

While our study did not identify the specific functional components within mEVs responsible for protecting BOECs from OS, the findings suggest that surface proteins and RNA may not be the primary mediators of their protective effects in the context of our experimental model. Instead, internal cargo, including miRNAs and proteins, may potentially play a dominant role. Our findings suggest that different classes of mEV cargo may contribute to the observed effects through distinct mechanisms. The miRNA analysis points toward pathways involved in post-transcriptional regulation and stress-responsive signaling, whereas the proteomic profiles highlight enrichment of peptidase-related and vesicle-associated pathways. Overall, the evidence indicates that the combined action of miRNA and protein cargo underlies the protective effects of mEVs in BOECs.

The current study provides reproducible evidence for the functional impact of milk-derived EVs on BOECs under oxidative stress, assessed through cell viability, *DDIT4* and *HIF1A* expression. In addition, mEVs promoted BOEC migration, further supporting their role in maintaining cellular function. Our primary aim was to demonstrate that the impact of milk-derived EVs can be detected at the transcript level. While detailed antioxidant profiling and mechanistic approaches, such as pathway inhibition or miRNA knockdown, would provide strong evidence for explaining the mode of action of mEVs, such investigations remain an important direction for future studies. In the present study, it has been determined that milk EVs exert a functional effect in protecting BOECs from oxidative stress, thereby establishing the basis for future mechanistic investigations. Additional characterization to delineate EV-specific mediators of these effects will therefore remain an important direction for our ongoing and future research. In this study, particle concentrations were selected within commonly used experimental ranges for milk EV research [80,81]. Also, dose standardization was performed using particle concentration. Nevertheless, the protein content of the enriched EVs was quantified to support dose transparency, revealing that a concentration of 10^9^ particles/mL corresponded to 0.5 µg protein/mL. However, treatment dosage may not be directly translated to in vivo conditions. Future work using more physiologically relevant dosages with animal models will be required to define meaningful dose–response relationships in vivo.

Beyond bovine in vitro systems, the findings of this study suggest potential applications of mEVs in reproductive health. OS is a recognized contributor to infertility, implantation failure, and early pregnancy loss [44,82]. Therefore, mEVs, which are resistant to gastrointestinal digestion [83] and enriched in bioactive cargos [84], may offer a non-invasive strategy to mitigate such effects. Future studies in rodent or bovine models of oviductal stress will be critical to evaluate biodistribution, safety, and reproductive outcomes. These approaches could provide mechanistic insights and translational evidence for the application of mEVs in human reproductive medicine.

## 5. Conclusions

In conclusion, this study highlighted the ameliorating effects of mEVs in mitigating OS-induced insults in BOECs. Using a resazurin-based viability assay, we demonstrated that mEVs significantly enhanced cell viability when administered before, during, or after CoCl_2_ exposure. The tropism of this protective effect was confirmed by the lack of aEVs to protect BOECs against CoCl_2_-induced cellular damage, emphasizing the unique properties of mEVs. Furthermore, a considerably higher impact of mEVs than the aEVs on inducing cell migration was detected. Lyophilization proved to be an effective storage method, as lyophilized EVs retained both their physico-chemical characteristics and bioactivity comparable to frozen EVs. Experiments using surface-modified mEVs revealed that the functional components responsible for this effect may play a minor role compared to the internal cargo. In silico analysis of common microRNAs, together with LC-MS/MS-based proteomic profiling of mEVs, indicates that these components are likely the principal drivers of the observed recovery effects. These findings contribute to the growing body of evidence supporting the potential of mEVs in OS-related therapies and encourage further research to identify the key bioactive molecules responsible for their protective function.

## Figures and Tables

**Table 1:** Primer sequences used for RT-qPCR analysis.

Gene	Primer Sequence (5′-3′)
DNA damage inducible transcript-4 (***DDIT4***)	Forward: GCTCGGACTGCGAATCCC Reverse: TCCAGGTATGCAGAGTCTTCCTC
Beta-2-microglobulin (***B2M***)	Forward: CTGCAAGGATGGCTCGCTT Reverse: GAATCTTTGGAGGACGCTGG
TATA binding protein (***TBP***)	Forward: GCACAGGAGCCAAGAGTGAA Reverse: TCCCCACCATGTTCTGAATCT
Hypoxia-inducible factor-1A (***HIF1A***)	Forward: GAGCCTGATGCTTTAACTTTGC Reverse: GAGTTTCAGAGGCAGGTAATGG

**Table 2:** Summary of key pathways and functional implications associated with common mEV miRNAs.

Enriched Pathways	Category	Relevance to Oxidative Stress & BOEC Recovery	Supporting References
3′-UTR–mediated mRNA destabilization	Post-transcriptional regulation	Modulates stress-responsive transcripts and supports restoration of homeostasis.	[37,38]
Telomere capping	Genomic stability	Oxidative stress is linked to telomere shortening and genomic instability; miRNAs may help maintain telomere protection.	[37,38]
Regulation of cytokine production	Inflammation	Consistent with reports of mEV-induced increases in anti-inflammatory and reduction in pro-inflammatory cytokines.	[39]
Negative chemotaxis	Cell migration	May promote protective migration away from harmful stimuli and support epithelial recovery.	[40,41,42]

## Data Availability

All the information that supports the findings of this study is available from the corresponding author upon reasonable request. The mass spectrometry proteomics data have been deposited to the ProteomeXchange Consortium via the PRIDE [85] partner repository with the dataset identifier PXD071883.
